# A Simulation-Integrated Approach to Advancing Pediatric Emergency Preparedness in Morning Report Within Residency Training

**DOI:** 10.7759/cureus.113321

**Published:** 2026-07-24

**Authors:** Kei U Wong, Harsh Sule

**Affiliations:** 1 Emergency Medicine, Rutgers New Jersey Medical School, Newark, USA

**Keywords:** emergency preparedness, experiential learning, low-fidelity simulation, medical education, pediatric emergency medicine, residency training, simulation-based education

## Abstract

Background

Pediatric emergency care presents unique challenges, and emergency medicine (EM) residents often have limited exposure to high-risk pediatric emergencies and resuscitation.

Traditional didactic methods may not fully bridge the gap between knowledge and clinical practice. With recent proposed revisions by the Accreditation Council for Graduate Medical Education (ACGME) and the American Board of Emergency Medicine’s (ABEM) shift toward simulation-based assessment, practical pediatric training strategies have become increasingly essential. This study describes the design, implementation, and learner perceptions of a low-fidelity pediatric simulation curriculum, defined as the use of basic mannequins and case-based scenarios without advanced physiologic modeling, embedded within morning report. Integrating brief, low-fidelity pediatric simulations into morning report offers a scalable approach that provides repeated exposure while addressing barriers to traditional simulation, including limited space, scheduling constraints, and resource demands.

Objective

To describe and evaluate learner perceptions of an educational innovation integrating brief, low-fidelity pediatric simulation into EM residency morning report.

Methods

From September 2021 to May 2025, a four-year EM residency program incorporated 27 pediatric simulation sessions into weekly pediatric-focused morning reports. Morning report is a required, in-person, faculty-led, 30-minute daily case-based conference attended by residents, rotating learners, and faculty, with concurrent clinical coverage. Required resources were minimal, relying on existing conference time, low‑fidelity equipment, and faculty facilitation. Sessions used low-fidelity mannequins and a portable simulation setup, along with web-based or scripted case scenarios with evolving clinical data, followed by facilitated debriefing. Participants completed post-session surveys assessing perceived knowledge, comfort, and teamwork; agreement proportions were analyzed using binomial tests.

Results

Of 194 participants, 138 (71%) completed surveys. Respondents included EM residents (103, 75%), medical students (15, 11%), attending physicians (12, 9%), and other healthcare professionals (8, 5%). Most respondents selected “agree” on the Likert scale, reporting perceived improvement in pediatric knowledge (126, 91%), comfort (121, 88%), and teamwork (125, 91%). The proportion of participants reporting agreement for each outcome significantly exceeded a neutral (50%) distribution (all p < 0.001). Additionally, 126 respondents (91%) indicated “agree” when asked about the continued incorporation of pediatric simulation into morning reports. When asked whether they would recommend the sessions to a colleague using a five-point Likert scale, 110 respondents (80%) reported “strongly agree,” and 15 respondents (11%) reported “agree.”

Conclusions

Low-fidelity pediatric simulation embedded within morning report was feasible, well accepted, and associated with perceived improvements in knowledge, comfort, and teamwork. This scalable approach may enhance pediatric preparedness while aligning with evolving graduate medical education requirements.

## Introduction

Pediatric emergency training is an integral component of emergency medicine (EM), yet residents have varied exposure to pediatric resuscitation and procedures across training sites. Traditional didactic teaching alone may not be an effective support for real-time clinical applications. Simulation-based education has been shown to improve learner confidence, procedural skills, interprofessional communication, and teamwork [1-3]. However, high-fidelity simulation often requires substantial resources, including dedicated space, specialized equipment, and faculty time. These constraints make high-frequency practice difficult to sustain, particularly in resource-limited or community-based programs [4-5].

Low-fidelity simulation, defined as the use of basic task trainers and structured case scenarios without advanced physiologic feedback, offers a pragmatic alternative that can be integrated into existing educational structures. This approach may be particularly useful in settings with limited access to simulation centers or constrained scheduling, while addressing gaps in pediatric exposure that are often sporadic and unpredictable across rotations for EM residents. Previous work in low-resource settings has shown that low-fidelity and in situ simulation can effectively strengthen pediatric emergency skills when paired with structured facilitation and debriefing [6].

Recent changes in the U.S. graduate medical education and board certification requirements further underscore the need for scalable pediatric educational strategies. With the proposed revisions to the Accreditation Council for Graduate Medical Education (ACGME) [7] requirements for EM programs to expand pediatric training exposure in EM, and the American Board of Emergency Medicine (ABEM) incorporating simulation-based assessment into its updated Certifying Exam [8], there is an increasing national emphasis on structured, experiential pediatric learning within EM training programs.

This study describes the development, implementation, and evaluation of learner perceptions of a low-fidelity pediatric simulation curriculum.

## Materials and methods

Design setting

The curriculum was implemented within a four-year EM residency program at a large urban academic medical center. Morning report is a required, in-person, faculty-led, 30-minute daily case-based conference attended by residents, rotating learners, and faculty, with concurrent clinical coverage. Required resources were minimal and relied on existing conference time, low-fidelity equipment, and faculty facilitation. Low-fidelity pediatric simulation scenarios were incorporated into approximately one pediatric-focused morning report session per month, depending on conference scheduling.

Curriculum development and case selection

Curriculum development was guided by Kern's Six-Step Approach, which includes problem identification, needs assessment, goals and objectives, educational strategies, implementation, and evaluation. Cases were developed and selected by pediatric emergency medicine (PEM) faculty, based on identified gaps in pediatric clinical exposure and educational priorities within the residency program [9]. To enhance feasibility and scalability, cases were adapted from established, publicly available resources (e.g., ACEP SimBox [10]), including facilitator guides and video-based scenarios with evolving clinical data, thereby minimizing development time and cost.

Topics emphasized high-risk, low-frequency pediatric conditions aligned with board-relevant content and clinical practice needs, including pediatric seizure, anaphylaxis, non-accidental trauma, newborn resuscitation, supraventricular tachycardia, sepsis, respiratory distress, blunt abdominal trauma, toxidrome, status asthmaticus, and infectious cases such as measles.

Simulation format, participants, and facilitation

Simulation sessions were conducted within the existing morning report conference space as 30-minute, case-based exercises. Each session utilized either a scripted scenario or a web-based video platform [10] to present a structured case with progressively evolving clinical information. Low-fidelity mannequins or basic task trainers were used in combination with readily available emergency department equipment, including a standardized pediatric equipment box, to enhance realism while maintaining a low-resource model.

Participants included medical students, resident physicians, attending physicians, and other healthcare professionals (including physician assistant students, nurse practitioner students, and ED pharmacists) rotating in the department. Average attendance per session was consistent with routine morning report participation (approximately 8-10 learners), although attendance was not formally recorded for each session. Scenarios were typically led by junior learners serving as the designated team leader, responsible for directing the clinical approach, assigning roles, and verbalizing decision-making throughout the case. Sessions were conducted in interprofessional teams, allowing junior learners to practice leadership, communication, and task delegation in a structured environment. Senior residents and faculty provided real-time support as needed, offering prompts or clarification to maintain case progression while preserving the junior learner’s leadership role. This structure was designed to simulate real clinical team dynamics while providing a psychologically safe environment for skill development and guided learning.

Each session concluded with a focused, faculty-led debrief that emphasized principles of clinical reasoning, communication, and teamwork. Facilitators were board-certified PEM physicians with experience in clinical teaching who reinforced key clinical and teamwork principles during debriefing.

Survey design

Participants completed anonymous, voluntary post-session surveys via a QR code immediately following each session. Survey items were developed by faculty for this curriculum and were not previously validated. The survey assessed participants' perceived improvement in pediatric knowledge, comfort in managing pediatric emergencies, teamwork and communication, and likelihood of recommending the instructional model to colleagues. This evaluation aligns with Kirkpatrick Level 1 (learner reaction) and Level 2 (self-reported learning) and does not include objective measures of performance or patient outcomes.

Responses were collected using a three-point Likert scale (Disagree-Neutral-Agree) for most items, while the recommendation question used a five-point Likert scale (Strongly Disagree-Strongly Agree). A sample post-session survey instrument is provided in Appendix A.

Data analysis

Descriptive statistics were used to summarize participant characteristics and survey responses. Categorical variables are reported as frequencies and percentages. For selected analyses, Likert responses were dichotomized into “agree” versus “neutral/disagree” to facilitate comparison of response distributions. For these dichotomized outcomes, binomial tests were used to evaluate whether the proportion of participants reporting agreement exceeded an expected proportion of 50%. All statistical tests were two-sided, and a p-value of <0.05 was considered statistically significant. Statistical analyses were performed using GraphPad QuickCalcs (GraphPad Software; Boston, MA, USA).

## Results

A total of 27 sessions were conducted between September 2021 and May 2025. Sessions were scheduled based on curricular availability. Of 194 participants who attended the simulation-enhanced morning report sessions during the study period, 138 (71.1%) completed the surveys.

Among respondents, EM residents comprised 103 (75%), medical students 15 (11%), attending physicians 12 (9%), and other healthcare professionals 8 (5%), including physician assistant students, nurse practitioner students, and emergency department pharmacists. No identifiable demographic data were collected. The project met institutional criteria for exempt educational research because it involved anonymous evaluation of an educational intervention and did not collect identifiable participant information.

Perceived educational impact

Participants demonstrated consistently positive responses. Using a three-point Likert scale (disagree-neutral-agree), 126 (91%) participants selected “agree” for improved knowledge of pediatric acute care, 121 (88%) reported greater comfort in managing pediatric emergencies, and 125 (91%) reported enhanced teamwork and communication following these sessions. The proportion of participants reporting agreement for each outcome significantly exceeded the expected proportion of 50% (all p < 0.001, binomial test).

**Figure 1:**
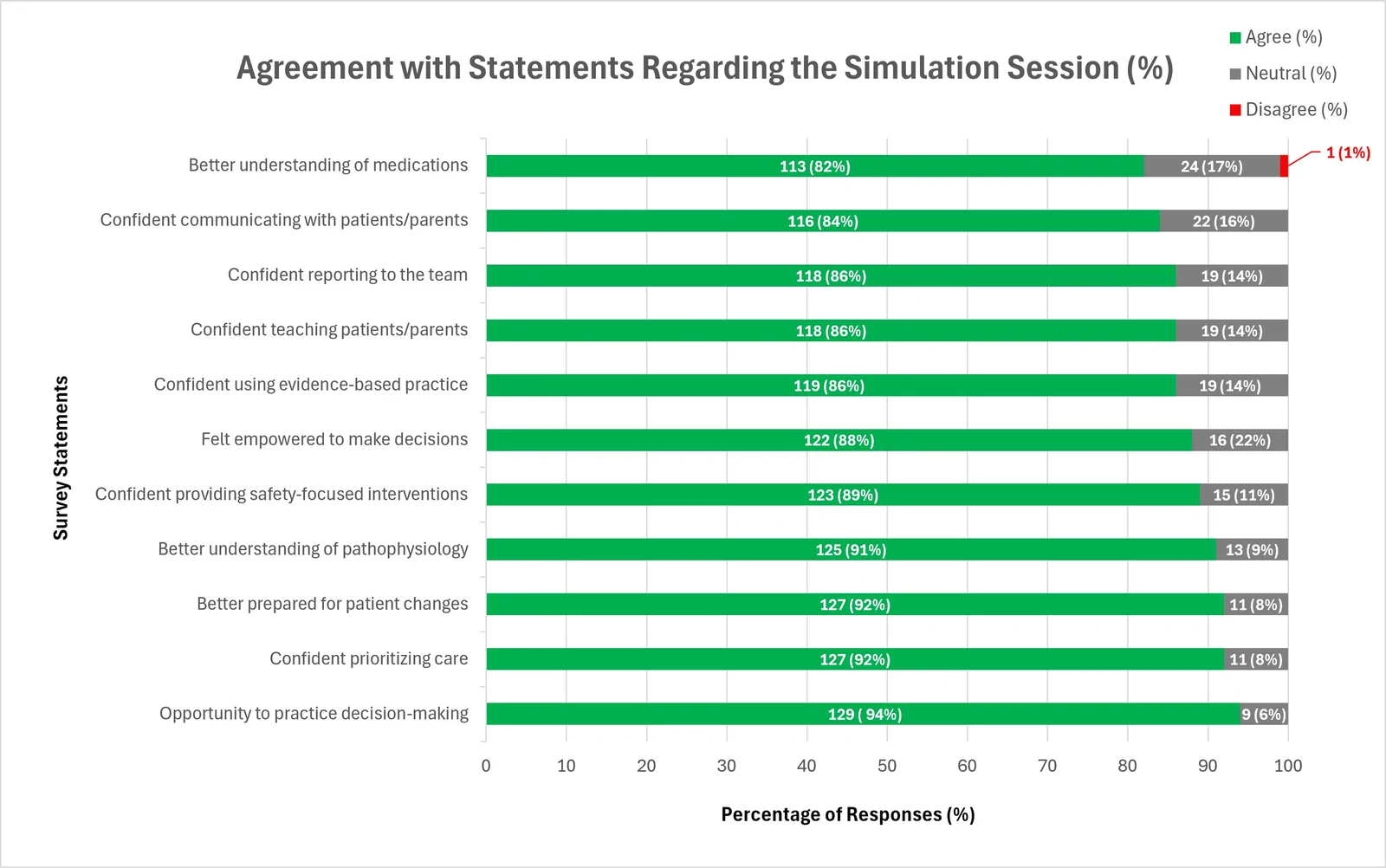
Learner Agreement With Educational Impact Learner perceptions of the educational impact of the simulation-enhanced morning report sessions. Bars show the distribution of responses, expressed as percentages, across the Agree, Neutral, and Disagree categories for each statement. Data labels are presented as n (%). Total respondents = 138. Agreement exceeded 50% for all items (all p < 0.001, binomial test). The figure was created by the authors using Microsoft Excel (no artificial intelligence (AI) tool or AI-assisted image-generation software was used to create it).

Detailed item-level responses are presented in Figure 1. Agreement was consistently high across all domains. The highest levels of agreement were observed for opportunity to practice decision-making (129, 94%), confidence in prioritizing care (127, 92%), and preparedness for patient changes (127, 92%). High levels of agreement were also reported for understanding of pathophysiology (125, 91%) and confidence in providing safety-focused interventions (123, 89%). Agreement exceeded a neutral (50%) distribution for all Figure 1 items (all p < 0.001, binomial test).

Domains related to communication and teamwork also demonstrated strong agreement, including confidence in communicating with patients and caregivers (116, 84%) and reporting information to team members (118, 85%). Lower, though still substantial, agreement was observed for understanding of medications (113, 81%).

Feasibility and acceptability

Acceptability of the intervention was high. A total of 126 respondents (91%) agreed that pediatric simulation should be incorporated into morning report on an ongoing basis. When asked whether they would recommend the sessions to a colleague using a five-point Likert scale (Strongly Disagree-Strongly Agree), 110 (80%) reported “strongly agree” and 15 (11%) reported “agree.”

Qualitative feedback

Narrative feedback during debriefings emphasized the value of practicing high-stakes pediatric scenarios in a low-pressure environment and highlighted simulation's role in reinforcing recent didactic content. Participants frequently described the sessions as highly valuable for improving clinical reasoning, structured approaches to patient care, and interprofessional communication.

Qualitative feedback from survey responses reflected strong enthusiasm for the simulation-enhanced morning report sessions, including several comments such as “excellent” and “extremely helpful." Learners valued junior-led scenarios with senior support and the realism of using actual pediatric ED equipment. They reported improved insight into clinical reasoning, structured approaches, and team communication.

Participants suggested enhancements, such as incorporating post-session summaries and expanding procedural practice opportunities. Overall, the sessions were perceived as highly valuable and effective for improving pediatric emergency preparedness. Detailed qualitative feedback themes are summarized in Appendix B.

## Discussion

Pediatric emergency care remains a challenging domain for EM trainees, given variability in clinical exposure. In the United States, approximately 30 million children visit emergency departments annually, with more than 80% receiving care in general emergency departments, underscoring the need for pediatric preparedness across diverse clinical settings [11].

While the role of the EM physician in caring for children will continue to expand, EM graduates often report feeling unprepared or apprehensive when managing pediatric patients after completing their residency [12-13]. In one study, EM program directors have also reported lower confidence in their graduating residents’ preparedness to care for pediatric patients compared with adult patients [14].

National commentary on the proposed revisions to the ACGME EM requirements underscores both the increased emphasis on pediatric emergency care training and the ongoing challenges of ensuring adequate clinical encounters, particularly for children under 12 years and neonatal resuscitation, given the variable pediatric patient volumes, uneven access to pediatric centers, and unpredictable case mixes across training sites [15]. This increased focus reflects longstanding evidence that younger pediatric patients, especially neonates and young infants, are among the most anxiety-provoking for EM physicians [16-17]. Recent position statements echo these concerns, calling for practical, flexible approaches and highlighting the need for scalable educational strategies that can reliably supplement limited pediatric exposure [18].

Interactive case-based teaching sessions, such as morning reports, have long served as a model for residency education. When paired with simulation and structured debriefing, these sessions provide immersive practice that translates didactic content into applied clinical reasoning, addressing a common gap caused by sporadic pediatric exposures across training sites. By leveraging existing conference time and minimal equipment, this model provides a feasible and scalable approach to expanding experiential pediatric learning across diverse training environments.

In this study, integration of low-fidelity simulation into morning report was associated with high levels of perceived benefit across multiple domains, with 88-91% of participants reporting improvements in knowledge, comfort, and teamwork. These proportions significantly exceeded a neutral distribution (all p < 0.001), supporting that observed effects were unlikely due to chance. Although high-fidelity simulation may provide greater realism for selected scenarios, low-fidelity approaches have been shown to achieve meaningful educational outcomes when supported by effective case design and debriefing.

Our findings suggest that brief, low-fidelity simulations integrated within the morning report are resource-efficient and perceived as beneficial for reinforcing pediatric competencies. This educational approach is also practical and scalable. Low-fidelity materials were sufficient to facilitate rich discussions and active learning, reducing dependence on simulation center infrastructure and minimizing barriers related to space, faculty availability, and scheduling complexity. These characteristics make the model readily adaptable across diverse EM residency programs, and particularly advantageous for community-based programs without access to high-fidelity simulation facilities, limited access to dedicated pediatric centers, or variable pediatric volumes.

Item-level analysis further demonstrated consistently high agreement across domains such as clinical decision-making, prioritization, communication, and preparedness, with agreement exceeding 50% for all measured items (all p < 0.001). These findings suggest that the educational impact extends beyond general perceptions to specific competencies relevant to pediatric emergency care. This study expands on existing literature by describing the integration of low-fidelity simulation into an existing educational conference structure, rather than as a stand-alone simulation session. Previous studies have examined low-fidelity and in situ simulation in both resource-limited global settings and U.S.-based programs; however, fewer reports have described embedding simulation into routine didactic sessions such as morning report.

Although this curriculum provided repeated exposure to pediatric scenarios over time, it did not employ a formal spaced repetition methodology characterized by structured intervals and deliberate case revisitation. While this curriculum was not designed or evaluated as a spaced-repetition intervention, it highlights a potential framework for future studies examining the effects of structured longitudinal reinforcement. Spaced training has been consistently supported in the medical education literature as an effective strategy to enhance skill acquisition, long-term retention, and transfer of learning compared with massed practice. Systematic reviews of simulation-based spaced education demonstrate improved learning and retention, particularly for procedural and high-acuity skills [19], and prospective cohort data further support sustained gains in knowledge retention with spaced approaches [20].

The recurring nature of these sessions suggests a potential opportunity to incorporate spaced-repetition principles in future curriculum development, although this hypothesis was not directly evaluated in the present study. Even without formal spacing, repeated reinforcement of pediatric concepts aligns with evidence demonstrating improvements in learner engagement, confidence, and retention [21]. Embedding these sessions within routine educational conferences also provides a psychologically safe environment to rehearse high-acuity, low-frequency skills that are particularly vulnerable to decay.

Limitations

This study has several limitations. As a single-site implementation within an academic EM residency, generalizability may be limited. Outcomes were based on self-reported survey data and are therefore subject to response bias. The survey instrument was locally developed and not previously validated. No objective measures of knowledge acquisition, clinical performance, or long-term retention were collected, limiting assessment to learner perceptions. Because attendance was not formally tracked for each session, precise participation rates could not be determined, and the potential for selection bias cannot be excluded.

Additionally, session frequency varied over the study period, with 27 sessions conducted over 45 months, reflecting competing curricular demands rather than a standardized delivery schedule. Formal feasibility outcomes, including implementation time, faculty workload, and cost analyses, were not collected. Feasibility was assessed descriptively based on successful integration into existing conference time using minimal educational resources. Furthermore, while statistically significant improvements in perceived outcomes were observed, these findings reflect self-reported measures rather than objective performance outcomes.

Implementation of this curriculum relied on an established morning report structure with protected educational time, which may not be available in all training environments, and sessions were facilitated by PEM-trained faculty, which may limit reproducibility in programs without subspecialty expertise. However, use of standardized, publicly available case resources may support adaptation by general EM faculty when paired with structured facilitation and debriefing.

Future direction

Future work should incorporate objective outcome measures, including assessments of critical actions, simulation-based performance metrics, pre- and post-intervention knowledge testing, and evaluation of long-term retention and transfer to clinical practice. In addition, future steps should explore whether integration of structured spacing or longitudinal reinforcement further enhances statistically significant improvements in learner outcomes.

## Conclusions

This study describes the design and implementation of a low-fidelity pediatric simulation curriculum embedded within an existing morning report structure. The model was feasible to implement within routine educational time and utilized minimal resources, making it a practical approach for programs seeking to increase exposure to high-risk pediatric scenarios.

Participants reported improvements in knowledge, comfort, and teamwork, with agreement proportions significantly exceeding a neutral distribution (all p < 0.001). While these findings reflect learner perceptions rather than objective measures of competence or clinical performance, this approach offers a scalable, sustainable framework that may support pediatric emergency preparedness and may help address variability in pediatric exposure across training programs, aligning with evolving U.S. training priorities, including ACGME pediatric requirements and ABEM’s incorporation of simulation-based assessment.

## Appendices

Appendix A 

**Table 1:** Post-session Survey Instrument This instrument evaluated participant demographics, case type, and perceived session impact across prebriefing, simulation, and debriefing domains using a three-point Likert scale, with an additional five-point scale for likelihood of recommending the session and an open-ended feedback component.

Section	Question/item	Response options/scale
Demographics	What is your role in the hospital?	Attending physician, resident physician, medical student, other (free text)
Session characteristics	What was the simulated pediatric case?	Newborn resuscitation, pediatric seizure, SVT, sepsis, anaphylaxis, respiratory depression, non-accidental trauma, other (free text)
Overall session impact	This session improved my knowledge of pediatric acute care	Disagree/neutral/agree
	This session improved my comfort in managing pediatric emergencies	Disagree/neutral/agree
	This session improved my psychomotor skills	Disagree/neutral/agree
	This session improved my teamwork and communication skills	Disagree/neutral/agree
Prebriefing	Prebriefing increased my confidence	Disagree/neutral/agree
	Prebriefing was beneficial to my learning	Disagree/neutral/agree
Simulation scenario	I am better prepared to respond to changes in a patient’s condition	Disagree/neutral/agree
	I am more confident communicating with patients/caregivers	Disagree/neutral/agree
	I am more confident prioritizing care and interventions	Disagree/neutral/agree
	I am more confident reporting information to team members	Disagree/neutral/agree
	I am more confident educating patients/caregivers	Disagree/neutral/agree
	I am more confident providing safe patient care interventions	Disagree/neutral/agree
	I am more confident using evidence-based practice	Disagree/neutral/agree
	I developed a better understanding of medications	Disagree/neutral/agree
	I developed a better understanding of pathophysiology	Disagree/neutral/agree
	I felt empowered to make clinical decisions	Disagree/neutral/agree
	I had the opportunity to practice clinical decision-making	Disagree/neutral/agree
Debriefing	Debriefing allowed me to verbalize thoughts before analysis	Disagree/neutral/agree
	Debriefing contributed to my learning	Disagree/neutral/agree
	Debriefing provided opportunities for self-reflection	Disagree/neutral/agree
	Debriefing was constructive	Disagree/neutral/agree
	Debriefing improved my clinical judgment	Disagree/neutral/agree
Recommendation	How likely are you to recommend this session to a colleague?	Strongly disagree/disagree/neutral/agree/strongly agree
Open feedback	Please provide feedback on your experience (suggestions, improvements, additional cases)	Free text

Appendix B 

**Table 2:** Thematic Analysis of Participant Feedback Thematic analysis of participant feedback from post-session surveys. Representative comments are presented verbatim and grouped into major themes reflecting learner perceptions of educational value, realism, leadership development, and areas for improvement.

Theme	Representative participant comments
High educational value and satisfaction	“Excellent morning report,” “Amazing case. We need more of these,” “Awesome sim!!! So helpful and informative!!” “Love these SIM sessions. VERY helpful for our learning,” “Great sim! Very educational,” “The best simulation I've been involved in,” “Great sim, always good to practice these,” “Very helpful.”
Experiential learning and realism	“Great case, difficult scenario so great to practice,” “This was a great way to practice rare situations in a safe environment,” “Simulation cases are very helpful to practice and gain confidence… in a low stakes situation,” “The sim was really well run… making the scenario seem very real,” “Having the airway box nearby… and having the vitals improve on the monitor… was helpful,” “Great practice! Much needed… actual airway kit and Broselow tape and real-time monitor.”
Improved confidence and preparedness	“Pediatric status is something that scares me immensely and running through this is so helpful,” “Every critical case… is anxiety provoking, and having these sessions help abate some of that anxiety,” “Extremely applicable case for an emergency situation that any of us would be uncomfortable with,” “Allows for immediate implementation of newly learned skills and further reinforcement of new clinical knowledge.”
Junior leadership and team dynamics	“Emphasis on juniors taking leadership roles is good,” “Team leader is a good idea,” “Hearing the thought processes of junior residents… allows me to reflect on how to coach them better,” “For overwhelmed juniors… acknowledge appropriate time to ask help from more senior colleagues,” “For seniors, work on supporting junior colleagues… to take more leadership roles,”
Clinical reasoning and structured approach	“Emphasis on schematic approach was well delivered,” “The discussion helps to formulate thinking patterns for next time,” “Helpful case… learned to consider arrhythmias in pediatric patients,” “Love the focus on ABCs,” “Would like to be questioned more on specifics… how much?”
Interprofessional learning and communication	“This was a great case to emphasize… communication skills among all members of the team,” “Practice/think about familiar cases in different ways,” “Recommend having someone focus on interventions and someone else act as the parent.”
Desire for more sessions and expanded content	“More cases,” “We need more of these,” “This should be more routine and scheduled on a weekly basis,” “Would love more cases generally,” “Would love a splinting… or cardiogenic shock case.”
Requests for supplemental materials	“Final discussed or printed bullet point summary,” “Providing relevant reading material to review after the sim,” “Provide handout or link with guideline management,” “Having a checklist would be helpful so we know what critical items we did or missed.”
Content diversity and representation	“Would love to see images of burn on brown and black skin to improve my clinical awareness.”
